# Civil inattention—On the sources of relational segregation

**DOI:** 10.3389/fsoc.2023.1212090

**Published:** 2023-09-05

**Authors:** Ilkka A. T. Arminen, Anna S. M. Heino

**Affiliations:** Faculty of Social Sciences, University of Helsinki, Helsinki, Finland

**Keywords:** civil inattention, conversation analysis, ethnomethodology, gaze behavior, interaction order, relational segregation

## Abstract

The article employs ethnomethodological conversation analysis (CA) and experimental video analysis to scrutinize the gaze behavior of urban passersby. We operationalize Goffman's concept of civil inattention to make it an empirical research object with defined boundaries. Video analysis enabled measurement of gaze lengths to establish measures for “normal” gazes within civil inattention and to account for their breaches. We also studied the dependence of gazing behavior on the recipient's social appearance by comparing the unmarked condition, the experimenter wearing casual, indistinctive clothes, to marked conditions, the experimenter wearing either a distinct sunhat or an abaya and niqab. The breaches of civil inattention toward marked gaze recipients were 10-fold compared to unmarked recipients. Furthermore, the analysis points out the commonality of hitherto unknown micro gazes and multiple gazes. Together the findings suggest the existence of subconscious monitoring beneath the public social order, which pre-structures interaction order, and indicates that stigmatization is a source for relational segregation.

## Introduction

The idea for this study originated when Ilkka was walking with a friend who has a medical condition that causes sensitivity to direct sunlight. During summer she has to cover herself with sun protection clothes, none of which are convenient, and all look alienating. Besides clinical sun protection clothes, she has used an abaya and niqab.^1^ When I was walking with her in the city center of [city] and she was wearing a niqab, I felt that she (or we) attracted some exceptional gazes, including half-hidden looks toward her/us. As vivid as the experience was, I was not certain to what degree I just made it up. An idea for a study started to evolve from that moment on.

Goffman (1963a, 1967, 1971) spent much of his career developing a new field of microsociology that explores the behavioral patterns through which the social order is created and maintained in everyday engagements. In line with then-evolving ethnomethodology, Goffman pursued a paradigm shift according to which human behavior in everyday life is not random but an orderly product. Social activities do not evolve from stochastic processes but are achievements based on actors' orientation. A key for order in public places is the distinction between “engagements” and unfocused, unratified, anonymous public behavior (Goffman, 1963a); for Goffman, what he calls “civil inattention” is a socially organized boundary mechanism through which regard without interest is allocated to unacquainted persons without sharing an invitation to become involved in engagement. Incessant maintenance of a distinction between those ratified to receive focused attention and others makes civil inattention both central to public order and enormously common. According to Goffman (1963a, p. 101), civil inattention is the most frequent interpersonal ritual.

The introduction of a new research object, orderliness of public behavior, was in part coined with the help of a subtle concept of civil inattention that refers to a dual-edged ritual through which appreciation is granted to a recipient without allowing recognition (Goffman, 1963a, p. 84). In that way, a civil, auspicious order is maintained among non-ratified anonymous parties in the public order without giving reasons for engagement. Civil inattention is thus a delicate, artful practice, which refers to a behavioral pattern of giving a brief, unnoticeable glimpse during encounters: “In performing this courtesy the eyes of the looker may pass over the eyes of the other, but no ‘recognition' is typically allowed” (Goffman, 1963a, p. 84). Goffman explains, for “persons passing on the street, civil inattention may take the special form of eyeing the other up to ~8 feet [about 2.4 m], during which time sides of the street are apportioned by gesture, and then casting the eyes down as the other passes—a kind of dimming of lights” (Goffman, 1963a).

Returning to the ethnographic epiphany, the auspicious, anonymous order seemed fragile and vulnerable to breaches. This was already noted by Goffman, who did pay attention to breaches of order, including “stares” among the unacquainted and even “hate stares” (p. 83), or failures to reciprocate friendly gazes, called “cuts” (p. 115). Referring to J. H. Griffin, Goffman (1963a) considered hate stares as akin to what “a Southern white sometimes gratuitously gives to Negroes walking past him^2^”. In 1971, Goffman articulated a wider framework of primatology for gaze behavior, including dominance hierarchies, “character contests” (p. 16), and an extension to remedial exchanges. Systematic studies of gaze behavior in public places have remained relatively rare, yet some examples are discussed in the next section. Nor has there been a solid development of studies on the relationship of public behavior and relational segregation discussed by Gardner (1980), Collins (1981), Goffman (1983), or Giddens (1990, p. 81–82).

Accordingly, we designed a field experiment to test the gaze behavior of urban passersby between varying social group members in the [city] cityscape. The experiment involves several elements and aims. First, we wanted to empirically explore whether civil inattention is observable between unacquainted passersby. Second, we aim at making the lengths of gazes measurable to establish measures of “normal” gazes within civil inattention and breaches from that. Finally, following the ethnographic epiphany we include a comparative dimension to see whether civil inattention is dependent on the social category of the person viewed. To operationalize the social category, we dressed up the experimenter (always the same person) with three different sets of clothes: casual “unnoticeable” western clothing, a distinctive sun hat that covered the face, and an abaya and niqab. A pedestrian with casual western clothes indexed an ordinary passerby; a pedestrian with a distinctive sun hat^3^ indexed a deviance from normal appearances without any explicit symbolic content; and a pedestrian with abaya and niqab indexed a tie to an identifiable social group with a symbolic religious value (Tarlo, 2010; Almila, 2016). The pedestrian with casual clothes could be used to establish the standards of gaze behavior among unmarked pedestrians in the [city] cityscape. With the help of marked choices of clothes, the dependence of civil inattention on social category was explored. In the data and methods section, we discuss the details of the experiment and the technologies utilized both in the experiment and in the analysis.

We will next examine the salience of gaze behavior for public order, and some attempts to empirically address the alleged phenomenon of civil inattention. We will then open the data and methods of our experimental research design, as well as its ethics. Our analysis concerns the measurability of public gaze behavior, empirical measures for the gaze in civil inattention and the types of breaches of normality. In the second part of the analysis, comparative measurements are utilized to determine the category boundedness of gaze behavior toward members of different social categories. In the discussion, we elaborate the empirical findings on the existence of civil inattention and the social determinants of breaches of civility. We close the discussion by expounding on civil inattention as a boundary mechanism that to some extent grants exclusive auspicious public order; passersby who deviate from normal appearances may not be granted the same level of approval and civility in public areas as those whose appearance confirms the local cultural norms. The analysis shows that the amount of uncivil attention follows categoric identification; the consequent relational segregation may form a basis for recognition disparity that hinders the participation of stigmatized groups to civic sphere.

### Civil inattention

Erving Goffman's studies of behavior in public places (Goffman, 1963a, 1967, 1971) addressed the patterns through which parties expressed respect to each other's need for personal space in otherwise crowded surroundings. In a modern cityscape, every individual daily passes a countless number of others, and sharing attention with everyone is simply impossible. Consequently, the passing of two individuals in the street should remain unfocused so that both parties maximally glance each other briefly while passing in and then out of view (Goffman, 1963a, p. 83–88).

In practice, a passerby walking down the street is constantly “scanning” an oval-shaped area ahead them, longer in the front and narrower on their sides, and briefly checking the individuals who are entering this area to avoid collision. If nothing alarming is detected, both interactants may feel at ease and turn their attention elsewhere (Goffman, 1971, p. 11–13). Inasmuch as the civil inattention thus formed may just be a conventional, routinized ritual, its breaches might be considered alarming (Goffman, 1971, p. 246–247). The closer one gets to the passerby, the more important the maintenance of civil inattention becomes. At a close distance, the exposure to possible staring grows (Goffman, 1963a, p. 84–85). As a ritual designed to maintain each other's personal space, civil inattention is a moral obligation between respectful individuals (Goffman, 1967).

Unfocused interaction, even a brief passing, also conveys information; individual appearances and gestures are modes of communication (Goffman, 1963a, p. 33–34). Goffman (1963a, p. 84) defines the function of civil inattention as follows: “one gives to another enough visual notice to demonstrate that one appreciates that the other is present … while at the next moment withdrawing one's attention from him so as to express that he does not constitute a target of special curiosity or design.” As civil inattention signals mutual respect and acceptance, it forms an implicit social contract between passersby and gains a normative weight. It is the key for mutual facework; each individual projects claims of self-approval and confirms the claims of others (Goffman, 1967, p. 105–106). It expresses acknowledgment of the other's presence and the absence of any fear, hostility, or avoidance toward the other (Goffman, 1963a, p. 84–85). Therefore, any breach of civil inattention—both by not looking or by staring openly—challenges the norms of public behavior. By neglecting patterns of respectful behavior, the individual withdraws from giving others signals of acceptance.

Soon after Goffman, Cary (1978) conducted experiments to see whether civil inattention as defined exists. The first two studies included the use of a hidden camera, which captured pictures of passings at a university campus. The results showed no distinct head movements that would support the existence of civil inattention; however, compared to present technology, the recording methods were insufficient. After Cary, more recent studies have supported the existence of civil inattention. Zuckerman et al. (1983) discovered that in elevators, most passengers looked at the experimenter once or twice, while the gazes remained relatively brief (median 0.35 s). Moreover, civil inattention was rated as the politest form of behavior. Hirschauer (2005) further confirmed the salience of civil inattention in elevators. Furthermore, Haddington (2012) showed how civil inattention is maintained even in exceptional situations, establishing a rule of the maintenance of polite distance on all occasions.

De Stefani and Mondada's (2018) video recordings of public encounters show how the transition from unfocused to focused interaction between unacquainted individuals is accomplished by adjusting both the trajectory and the bodily orientation toward the target individual. However, the shift away from civil inattention requires a verbal account, such as for example, asking directions (De Stefani and Mondada, 2018). Also, additional attention without entitlement is considered rude. Horgan (2020) examined breaches of civil inattention, which he coined “uncivil.” According to Horgan's interviews, uncivil encounters are not rare: over a quarter of the participants reported experiencing uncivility from an unacquainted person during the past week, and over a half during the past month. However, Horgan (2020) did not focus on gaze behavior: instead, his interest lays in more direct rude behavior, such as street remarks, bumping into someone without apologizing, cutting in line, or even threats of violence. We might expect that uncivil gaze behavior exists as well.

The concept of normality is a key for civil inattention; according to Goffman (1963a), an open stare is a signal that exposes undesirable attributes of the receiver, implying that they lack the right to receive civil inattention. In brief encounters between passersby, this evaluation is based on first impressions, the importance of which Goffman (1959, p. 22–24) highlights. Based on his studies, Goffman (1963a, p. 11) suggests that “fitting in” (i.e., following the behavioral patterns of the common public) seems to be primordial for any situation. Being “inappropriate” may lead to the individual being stared at, or alternatively neglected, or treated as a non-person. Both excessive attention and withholding of attention may be used as negative social sanctions. A similar duality is also found in extended gazes, which may signal positive attention, admiration, and interest (Mason et al., 2005). An experiment conducted by Patterson et al. (2010) shows how passersby display significantly more glances if gazed at first, and even more so if smiled at.

Civil inattention is maintained until something begs for extra attention, be it positive or negative. According to Goffman (1971, p. 239–247), normal appearances signal stability, giving the individual a chance to continue their own business without concern. But normality is also a moral requirement. When an individual breaks the limits of standard behavior, one may receive an “overlong look,” which suggests that corrective behavior is required. As for personal appearances, it “is usually the case that normal appearances, typical appearances, and proper appearances are much the same” (Goffman, 1971, p. 240). The visual presentation, then, affects how random passersby interpret each other merely by gazing. In addition to personal features, prejudices toward certain cultural or racialized appearances affect this treatment. It is possible that the performance of civil inattention varies, not only between different cultures (Watson, 1970; Rossano, 2013) but also depending on the expected social status of the receiver (Gobel et al., 2015). Gardner (1980) emphasizes that the norm of civil inattention differs significantly, depending on the gender of the other party: just like children or racialized persons, women are easy targets for both positive and negative attention. Patterson's (2005) empirical study of passings between unacquainted on a college campus concluded that female confederates received four times more gazes than males. Regardless of the reasons for the gaze, the target typically recognizes the extra attention and may aim at disguising themself to ensure the other that nothing untoward is taking place. As a result, self-aware normality will be performed (Goffman, 1971, p. 256–273).

### First impressions, clothing, and veiling

Human visual sensory mechanisms operate at an astonishing speed. According to Thorpe et al. (1996), it takes only 20 ms to pass a go/no-go categorization test (e.g., whether there is an animal in the picture) with 94% accuracy. Further, categorization of an object takes barely more time than detection; as soon as we notice something, we perceive what it is (Grill-Spector and Kanwisher, 2005). Willis and Todorov (2006) present evidence for rapid first impressions between persons. While 100 ms is sufficient for forming a first impression of a person, one additional second of evaluation time does not essentially change it. In more complex visual social clues combining gaze direction, pointing gesture, and emotion, all these signals are fully integrated at 200 ms (Conty et al., 2012). Behind all this is a human interaction engine; the average time lapse between turns in conversation is around 0–200 ms, with visual communication cues further speeding the processing of language (Hömke et al., 2017; Holler and Levinson, 2019; Levinson, 2020).

As a form of collecting information and organizing interaction accordingly (Rossano et al., 2009), gaze is also an important dimension of personal space. Due to the rapid visual system, ordinary encounters proceed smoothly without extended gazes. An impression that indexes a breach of normality calls for making a prolonged gaze, with the help of which abnormality is categorized and made manageable (Garland-Thompson, 2006). Impressions are holistic; they merge outlook, behavior, and visual characteristics, such as attire, into a categorical whole. Clothing works as an important type of non-verbal communication in conveying the social characteristics of passersby. Therefore, clothing may affect the gaze behavior between passers-by. Furthermore, the types of outfits worn may invoke related types of gaze behavior. El-Geledi and Bourhis (2012) found that students in Quebec assessed a person with Western clothes more positively than a person wearing a Muslim hijab, while a person with a niqab (face veil) scored even more negatively. Equally, Muslim women's veiling with either a hijab or a niqab was assessed negatively by British students (Everett et al., 2014). In Western media representations, burqas and other types of Muslim veiling are presented as oppressive (Rantanen, 2005). For the Dutch, face veils tend to evoke feelings of anxiety, fear, and even hate (Moors, 2009). In Finland, and presumably in many other Western countries, face veils paradoxically make women more visible, and they symbolize difference; veiled women get labeled Muslim above anything else (Karhunen, 2022). Finnish Muslims report themselves to be distinct from the Finnish majority and find it difficult to be both Muslim and Finnish (Pauha, 2018). Muslim veiling is also a way to strengthen one's identity category, although it may result in harmful miscategorizations by others (Hopkins and Greenwood, 2013). This is especially true for face-veiling. Almila (2016) describes wearing face-veiling in Finland as a form of resistance against prevailing social norms; however, it puts the person in a vulnerable position, being judged by the non-Muslim majority and assessed by other Muslims.

Although Muslim veiling has raised political debates in Western countries (Moors, 2009; Shirazi and Mishra, 2010), people who wear face veils are a small minority. For example, the estimate of the Finnish Muslim population was 120,000–130,000^4^ in 2022. Although percentages are growing in many countries, only a minority of Muslims wear traditional clothes, at least in Finland. Konttori (2022) estimated that only some hundreds of Muslim women in Finland wear niqabs.^5^ Also, the attitudes toward Muslim veiling have predominantly been negative in Finland. About 37% of Finns had a negative or very negative attitude toward a hijab, and 72% toward a niqab (Kirkon tutkimuskeskus, 2012, p. 51). Overall, Finns have rather negative attitudes toward Islam, also more negative than many other European countries (Martikainen, 2020; Karhunen, 2022).

In Western countries, one of the main public concerns regarding women wearing niqabs is the lack of emotional signals and facial recognition due to the lower part of the face being covered. Fischer et al. (2011) found that a viewer tends to interpret more negative emotions from a partly covered face, both in niqab and computer-altered control-case conditions. The negative interpretations then affect one's attitudes toward covering the face, which are also potentially strengthened by existing stereotypes. Tarlo (2010) has witnessed excessive staring toward women in niqabs, which is explained, for instance, by the “need to look harder to reassure yourself that there is a person under there” (Tarlo, 2010, p. 134). Moreover, according to Moors (2009), one of the reasons behind the discomfort caused by interacting with a person wearing a face-veil is the fact that “the face-veil itself enables them to see without being seen.” Based on these findings, a niqab not only reveals the affiliation but also disguises the gaze; in addition to negative stereotypes the lack of visible cues and interaction may cause discomfort.

### Data and methods

We conducted a field experiment to test the gaze behavior of urban passersby between members of varying social groups in the [city] cityscape. The data was collected during six 90-min afternoon sessions in late August and early September 2017. We used a hidden GoPro 5 video camera, which was attached to the experimenter's chest, to record and analyze the gaze behavior of random passersby. All the sessions took place in central [city] within a preplanned walking route. During these sessions, the experimenter wore three different outfits: (1) a regular Western outfit, (2) a face-covering sun hat paired with dark sunglasses, and (3) an abaya with a niqab. Of these three outfits, the regular Western outfit worked as a baseline of gaze behavior. The experimenter was instructed to behave “normally,” that is, not to intentionally avoid gaze contact but not to seek it either. Consequently, data cannot answer questions of reciprocity (i.e., whether extended gazes or an avoidance of gazes could be invoked). On the other hand, the data reflects uninvited departures from “normal” gazes. The recordings in total produced ~3 h of video data for each of the three different outfits. In addition, we had an assistant following and observing the situations during the recording sessions. This observation produced some notes, which were used to complement and assess our video data during the initial analysis.

**Figure 1:**
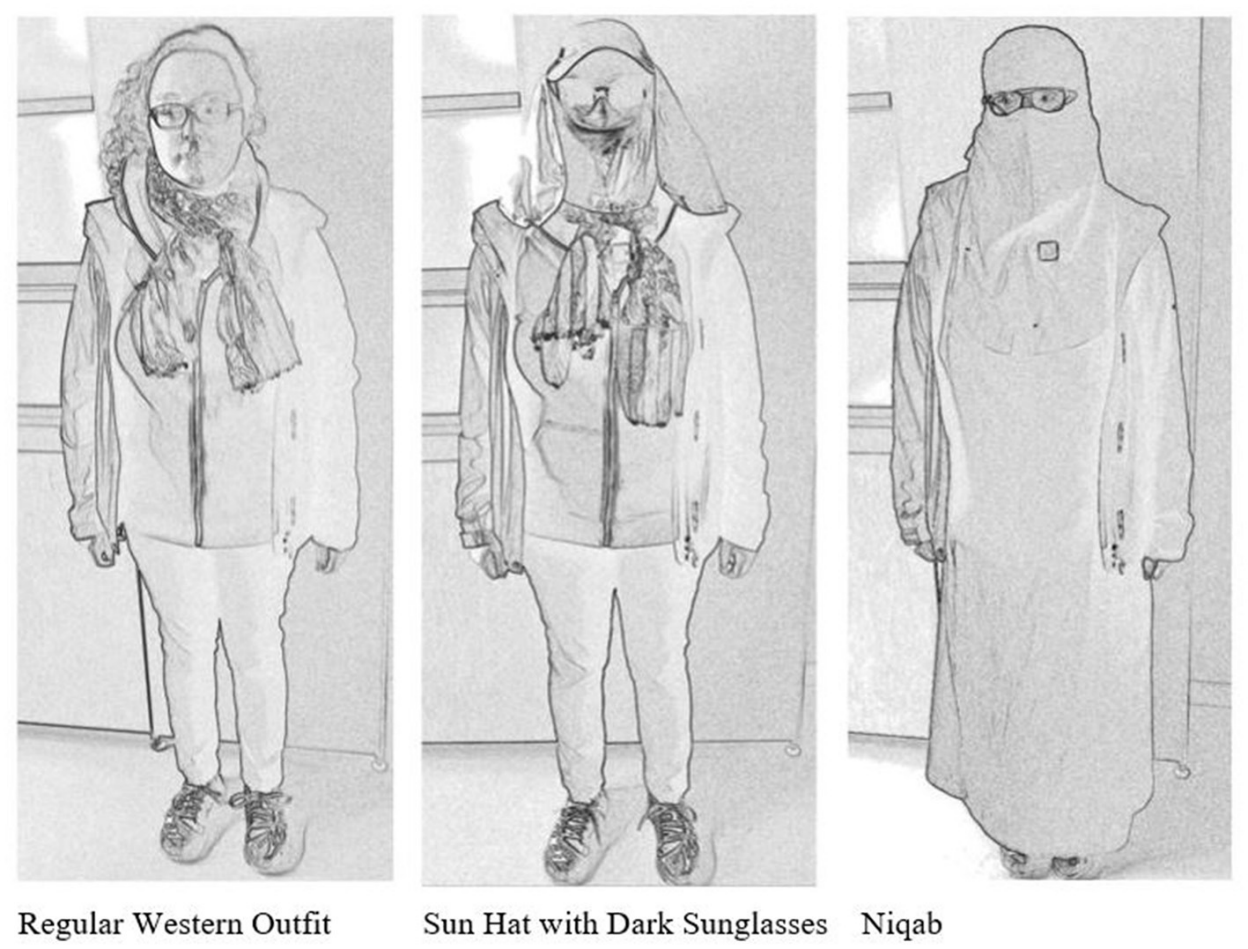
Experimenter with three different outfits.

Figure 1 presents each of the outfits. On the left, the experimenter is wearing her regular clothing, which does not stand out from the crowd in [city]. In the middle, she has the same outfit but combined with a face-covering sun hat and a pair of dark sunglasses (the anonymization by a negative picture may exaggerate the peculiarity of the sun hat). On the right, she is wearing a completely black outfit, an abaya with a niqab. The video camera is slightly visible in the last photo: an observable reader might detect a small square (the lens of the camera) on the experimenter's chest. In brief passings, it is unlikely that the camera could be detected. In both the sun hat and niqab outfits, the experimenter's face is covered, and even more so in the sun hat since the dark sunglasses hide the experimenter's gaze completely. However, unlike the niqab, the sun hat is not a symbol of any religious or ethnic affiliation.

Our research design is a mixed-methods study combining ethnomethodological conversation analysis (CA) and experimental video analysis. Ethnomethodology refers to the study of the ways in which people build meanings together. Following Goffman (1963a, 1967, 1971) work, we approach gaze behavior as meaningful interaction between the participants. Based on the ethnomethodological standards of analysis, we do not attempt to interpret the hidden intentions of the passersby. Rather, we focus our attention on the observable gaze behavior, also excluding facial or bodily gestures, which would only become relevant if the ecological huddle, ratified encounter, has been established. The gaze contact precedes the formation of the encounter, then allowing a wider variety of semiotic resources. Although there is no verbal interaction in our data, the interactions are multimodal: the gaze is a means of embodied interaction, as is the physical movement of the passersby in the surrounding environment. For the purposes of this study, the precise temporal organization of gazing is especially important. This embodied multimodality is accounted for in our transcripts, inspired by Mondada (2018) multimodal conversation analytical transcriptions.

Experimental video analysis allowed a quantitative approach. The quantity of all potential cases (i.e., direct passings between the experimenter and other pedestrians) was estimated to be around 700 per outfit. In addition, the data contains about as many non-valid cases, due to backlit or shaky footage, too-crowded places, passersby wearing dark sunglasses, children, and smartphone users; these were all excluded. After excluding the non-valid cases, 100 cases were randomly selected for each type of outfit (50 cases from each of the six sessions), with the total N being 300. Although the amount of included data is limited, it permits some statistical findings and is still analyzable qualitatively. The GoPro camera cut each recording into 10 clips, which we used as a loose structure for our sampling. As a result, the guideline was to pick cases as symmetrically as possible throughout the data (only excluding technically or otherwise non-valid cases). The selected cases include both single passersby and pairs or groups of people as well as people of varying age, gender, and ethnicity.

These selected cases were analyzed first by simply watching them multiple times. At this stage, we focused on general impressions, such as possible gazing and its duration. More detailed analysis was accomplished by utilizing the video analysis program Adobe Premier Pro, by means of which the exact duration of the gazes could be calculated with 0.01-s accuracy. The program allowed examination of cases as series of individual frames, thereby creating precise timelines for each clip. With maximum frame-per-second settings and an effective zooming tool, it is possible to define the exact starting and ending points of the gazes and gestures. Moreover, this method allowed us to detect subtle gazes from a further distance which exceeded the limits of ethnographic observation. We focused on finding out: whether the passerby gazes at the experimenter and, if so, how long, and whether there are multiple gazes during the passing. Distinctive head movements were also considered. Finally, the comparison between the gaze behavior for the three different outfits is based on statistical analysis.

The research practices follow the guidelines of the Finnish National Board on Research Integrity. Although consent to participate was not requested in advance, the research is justified and could not have been carried out if the participants were asked for their consent to participate in the research. Data collection did not cause damage or harm to the participants. An ethical approval statement can be given by the review board. Ethically, this is a unique situation since we collected video recordings of a large number of passersby who did not know that they were being filmed. Finnish law allows filming in public places without asking consent from the persons visible in the recordings. The research ethics are maintained by preserving the anonymity of each subject of our data and by focusing solely on the larger patterns of gaze behavior instead of the personal traits of identifiable passersby. We also excluded children from our data. The usage of a hidden camera method is justified by the unique data it offers; we were able to gather a large data set of natural social behavior in high detail and quality. This would not have been possible had the participants been aware of the filming. In addition, video analysis is very useful for studying aspects of micro interactions that tend to be taken for granted as the subjects are most likely not completely self-conscious therefore it may reveal aspects of behavior with potential causal relations more accurately than more conventional methods, such as interviews or researcher observation.

We begin our analysis by examining possible classes of gaze behavior; initially looking at brief gazes that may represent civil inattention, and then narrowing down the empirical limits of observable civil inattention to a class in between non-gazes and extended gazes. After accounting for the varieties of gaze behavior, we continue by quantifying the initially qualitative data; we then aim to operationalize possible classes of gaze behavior, starting from zero cases where there is no gazing during the encounter to extended gazing that exceeds civil inattention. Finally, the quantitative measurements enable comparisons between the different data sets and thereby estimate the causal relations between the visual appearance of the experimenter and the gazes she received (Arminen, 2009).

## Analysis

We start our analysis with a qualitative assessment of public gaze behavior and try to specify Goffman's notion of civil inattention. We begin with very brief gazes and explicate their variations. Continuing from these, we try to explicate what other types of gazing behavior exist in the streets. Among these, we discuss passings without gazing and then move on to more distinct types of gazes that could be considered breaches of civil inattention. Throughout this section, we utilize multimodal transcriptions with some anonymized stills. Here, our aim is to introduce the reader to various types of gaze behavior in practice. After the qualitative findings, we present the counts of gaze behavior types as an aggregated quantitative result of public gaze behavior.

### Civil gazes

As discussed above, according to Goffman (1963a), civil inattention is the prevailing ritual between unacquainted individuals passing each other in urban public places. Notably, Goffman (1983, p. 6) focuses on persons as vehicular entities, that is, human ambulatory units, thereby suggesting that gazes are environmentally coupled with embodied mobile activity, as Goffman's follower Goodwin (2007) might have put it. Perhaps the most detailed explication of civil inattention characterizes it as a brief glance during passing, given around eight feet and then ended as the eyes are cast down as the other passes (Goffman, 1963a, p. 84). Given that in passing both parties are moving in direction toward the other, the characterization allows to operationalize the duration of civil inattention as a movement (see also Patterson, 2005). The brevity of gaze seems to be set around two steps or less, as with two steps of both parties passing have progressed to proximity that would strengthen the intensity of the gaze maintained to the degree that it would no longer be felt as “civil” but as an intense glance that would transfer the parties beyond disengagement. Indeed, in our material we do have several cases of that type, which we will discuss later.

In the following example, a case of civil inattention between passersby is shown.^6^ The passerby casts a brief gaze at the experimenter after crossing the street and noticing the upcoming passing. The gaze remains brief, just about one step long, as can be seen from stills.^7^


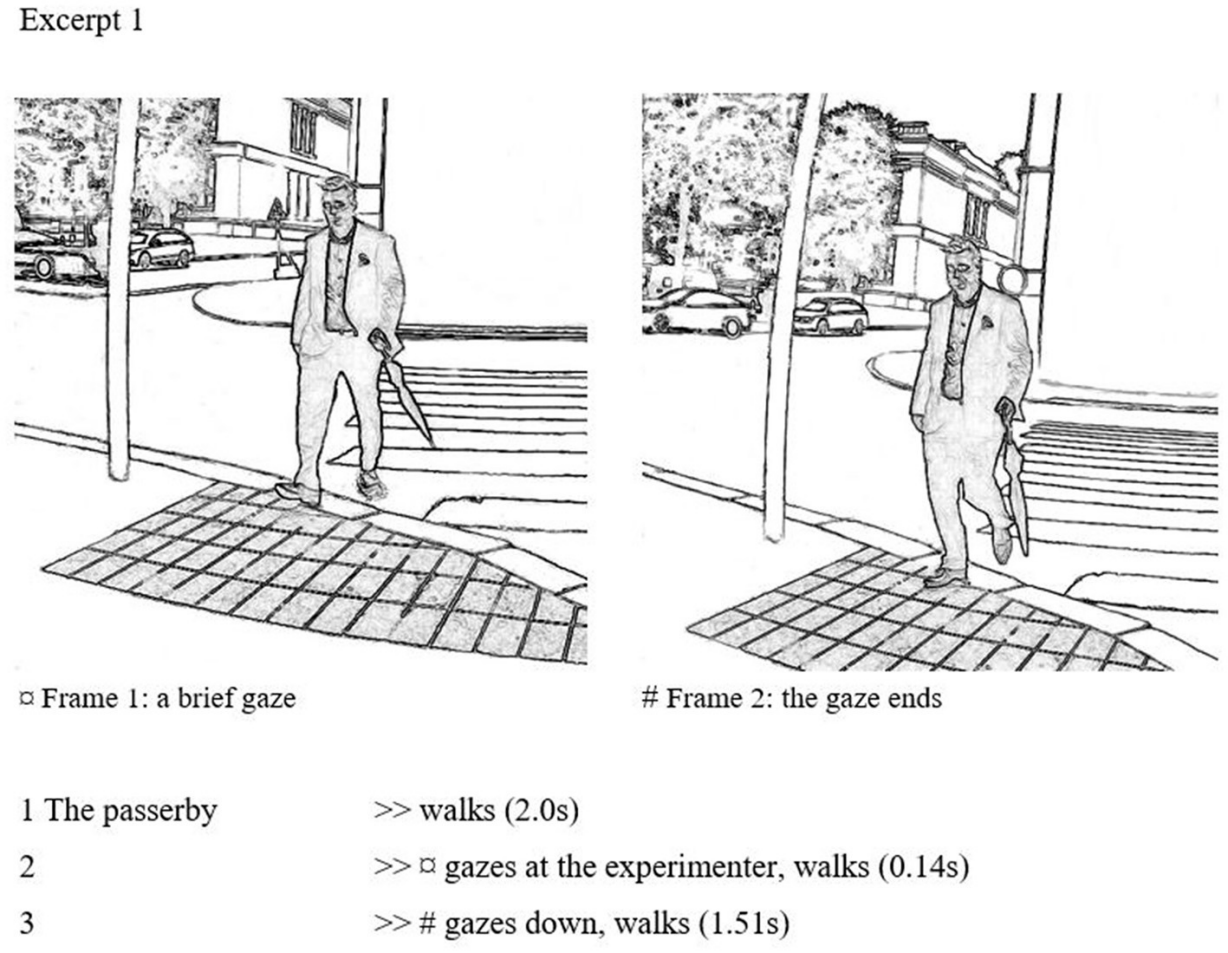


The passerby in this case approaches on a quiet street where both parties have visual access to the upcoming passing. In frame 1, the subject gazes briefly toward the experimenter. The duration of the gaze is very short. In frame 2, the passerby has already turned their gaze down. The precise distance between the passerby and the experimenter is hard to gauge, but the brief glance may have been initiated at a distance of roughly 2.5–3 m. The gazing takes place during a single step: in frame 1, the subject's front foot is taking a step, and in frame 2, the front foot has landed, and the back foot is starting to take another step as the gaze ends. This seems to be an archetypal case of civil inattention that matches well with Goffman (1963a, p. 84–85) characterization: it demonstrates that the passersby are scanning the surroundings as they proceed further and, as nothing causes alarm, the gaze is swiftly aimed down again. Anyhow, with the brief glance the passerby has communicated acknowledgment of the other, and by a rapid turning away of the gaze, the other's social space is recognized and intrusion into it is sanctioned by avoidance.

As noted also in the transcript above, our procedure allowed timing of gaze, which here is 0.14 s. According to Goffman (1963a, p. 84), “one gives to another enough visual notice to demonstrate that one appreciates that the other is present,” which would minimally require the other to be peripherally aware of the gaze behavior. Following Goffman (1981), Heath and Luff (1992), and vom Lehn et al. (2001) have explored peripheral awareness, that is, people's ability to process and utilize information of phenomena that are not in the focus but the periphery of attention. Very brief glances, or civil inattention, may allow a viewed person to be peripherally aware of the appreciation received, without having focused attention to the appreciation given. It may well be that 140 ms is not long enough for a focused mutual gaze contact, where a person had noticed another having noticed one's gaze, but it may be long enough to get a peripheral sense of a noticing, that is, someone having given a glance. In that way, brief glances may also establish the ritual nature of civil inattention, as Goffman suggested.

Although video analysis supports the existence of civil inattention, it also provides the basis for detailing, specifying, and elaborating it. First, the coupling of gait and gaze allowed us to consider a simple matrix of operationalization. Accordingly, at its shortest a brief glance of a pedestrian lasts less than a step. Indeed, the median length of our civil inattention cases is 60 ms, about half of the length of example (1), in which the gaze lasted almost a full step. In standard CA terminology (Sidnell and Stivers, 2012), cases that are shorter than a mini pause do not allow establishment of a mutual focused gaze exchange but may enable peripheral awareness. Two-step-long gazes (at a “normal” pace) can last up to 500 ms. They may be long enough for the other to notice another's noticing but still short enough to not yet to comprise a noticeable stare. Indeed, these longer glances may still be civil in the sense that the onlooker may have turned their gaze away after noticing that they were noticed.^8^ Therefore, the boundary of civil inattention might benefit from a closer look.^9^ Finally, it seems that gazing-aways after the “civil inattention” glance can vary. Goffman suggested that a gaze is closed by turning eyes down “a kind of dimming of lights” (Goffman, 1963a, p. 84). Empirically, an equally common pattern is to turn the gaze straight forward or completely away. Though Goffman's ethnographic insight and precision is admirable, ethnography has its limitations.

#### Modifications of civil inattention

A closer analysis of videotaped passings also revealed some aspects of civil inattention not discussed by Goffman. In many cases there are more than one gaze. Most likely, multiple gazes are not uncommon, and their detection depends on the observation methods used. If gazes are very short, <100 ms, they practically evade focused ethnographic observation; an ethnographer may become peripherally aware of them but remain unable to provide focused accounts of them. Very brief cases or series of them are not accountable; hence, Goffman did not discuss them. With the help of technical analysis, by exploring videos composed of series of individual frames, an analyst can pay attention to the minutiae of gazes that escape ethnography. In (2), the passerby gives two brief gazes, both of which are very short. On line 2, the passerby gives a gaze which lasts only 0.06 s. The gaze is barely noticeable, given the distance before passing. After the initial gaze, the passerby gazes away for almost a second, and then gazes again. The second gaze (line 4) is even briefer than the first one and could be characterized as a glimpse. During the actual passing, the passerby does not gaze at the experimenter.


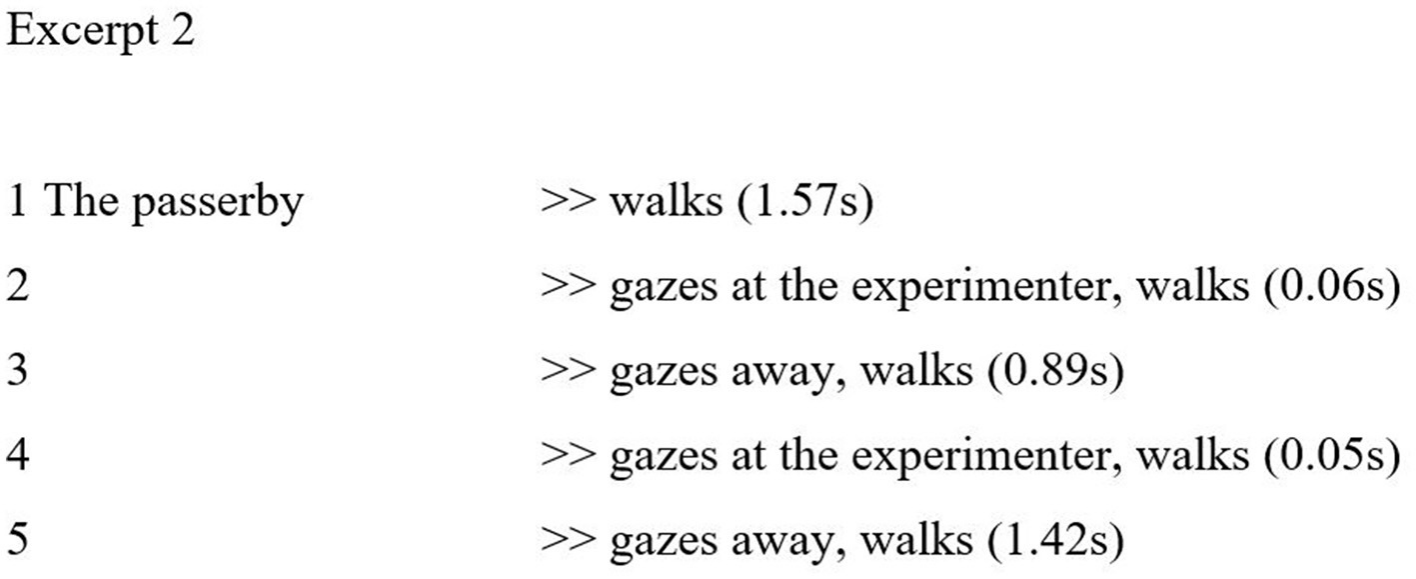


Both gazes in (2) are very brief and subtle, almost unnoticeable, and do not break the norm of polite distance. As (2) shows, both gazes are made from a distance with respect to the personal space of the experimenter; even after the second gaze, it still takes the passerby 1.42 s to pass the experimenter. The passing takes place on a relatively empty street, which makes the experimenter visually accessible already at a distance.

Although image identification can be reached as fast as 20 ms, more complex information-processing, combining aspects of an image, can easily take 200 ms (Conty et al., 2012). This would suggest that these very brief gazes, micro gazes as we would call them, are so short that the gaze does not seem to have stopped for a longer period; the image glanced appears not to have invoked a task of a more thorough exploration of the gaze object. These micro gazes may have been just a part of a pedestrian's routine scanning of the social scene, and they do not display greater involvement or interest. However, as with civil inattention more generally, these multiple gazes vary in length. Glances of up to half a second are different from micro gazes. Even when none of the multiple gazes are above 500 ms, the accumulation of more than one gaze may indicate more involvement than gazing once. If none of the gazes are no longer than 500 ms, the on-looker refrains from breaching civil inattention but commits themself to a particular gaze behavior. Gazing twice allows the passerby to gather more visual information than just one gaze, while not becoming openly impolite. We argue that the cases with two short gazes can be labeled as a subcategory of civil inattention; they are distinct from singular gazes but do not become engrossed in or seek engagement, and they maintain the spirit of civil inattention. Multiple gazes also stress the need for further studies, as the boundary between civil inattention and its breaches does not appear clean-cut.

### Passing without gazing

Civil inattention arises out of an ecology of involvements. It is a behavioral ritual to maintain auspicious public order without posing obligations to become engaged in interactions with unacqainted (Goffman, 1963a). The ecology of involvement includes structured practices of how involvements are allocated. In as much as a cityscape is composed of mobile individuals, there may happen millions of passings a day within an urban area (Giddens, 1990, p. 81). Moreover, mobility is a part of the ecology of involvements as it is itself a kind of involvement: mobile individuals are involved in getting somewhere, or away from somewhere. Of course, immobile pedestrians are also occupied with something. They may seem not to be doing anything, which may stand for doing as waiting; that is, they are occupied by their expectation of something (Ayass, 2020). Also, loitering can itself become an occupation, at least for street-corner gang members (Whyte, 2012). All involvements around which activities may become organized establish engagements that cut the parties away from the disengaged public order between detached individuals. Mobile pedestrians may have constraining involvements, too. Not untypically, people may be shielded by their engagement with smartphones (Ayass, 2014), but we excluded these cases of screen engagement from the data. Sometimes groups, or pairs, can be exclusively oriented to their conversations.^10^ Goffman (1963a) also discusses “aways” and occult involvements, when people are, so to speak, gazing inwards: passersby simply gaze down or keep the gaze seemingly unfocused, apparently lost in their own thoughts. Thus, involvements other than gazing passersby may become exclusive. Finally, there is also an economy of gazes. Gazes, like turns at talk (Sacks et al., 1974), are a sparse resource. A focused gaze somewhere means that it is away from elsewhere as a figure/ground distinction is made (Goodwin, 1994), and the ground is left with only peripheral attention. A busy cityscape poses a challenge to the economy of gaze, as it would be laborious to cast an equal gaze at every single passerby individually. Instead, peripheral attention may be the solution for the challenge of gaze resource limitations, and it provides one occasioned reason for a lack of gazes.

As civil inattention is based on peripheral awareness, and its civility, unobtrusiveness, makes its unaccountable, passings without gazes do not challenge the auspicious public order. All in all, it is not uncommon to pass passersby who do not cast the slightest gaze, even though they do not seem to be occupied with anything particular—or at least not in the way that would be decipherable from the recordings. During passing, a passerby may gaze straight ahead or slightly down, or alternatively focus their gaze elsewhere. In Excerpt 3, the passerby would have had plenty of time to gaze at the experimenter, but they look straight past her during the whole 3.63-s passing in a quiet street.


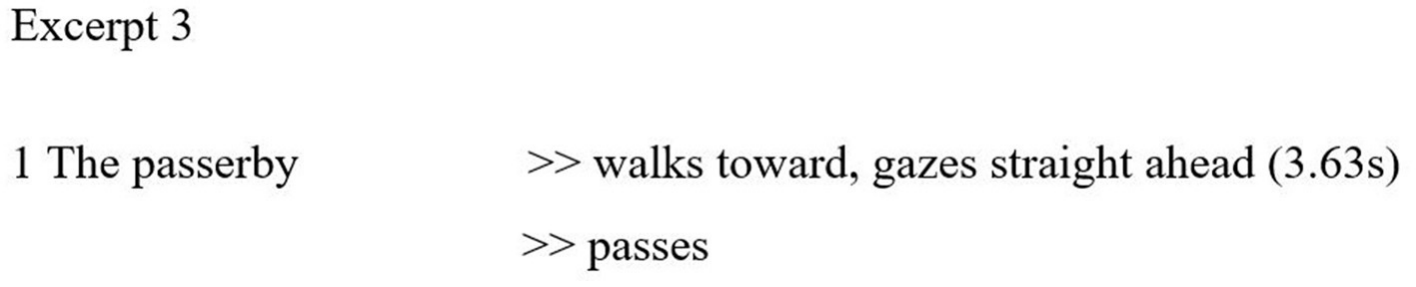


As such, passing without gazing is not accountable or noticeable. As an ethnographic note, neither the experimenter nor the assistant paid any extra attention to these passings; not receiving gazes from passersby feels perfectly normal. Not gazing seems to be taken for granted and is not taken to be accountable; it forms a part of the normal anonymous urban scene. It is also important to note that although the passerby is not gazing directly at the experimenter, their peripheral vision most likely captures her presence, allowing the passerby to adjust to the upcoming passing and navigate movements in a manner that displays recognition of personal space.

Not gazing is different from a concerted display of avoiding gazing. Goffman (1963a, p. 83) discusses non-person treatment, referring to practices that somebody is “not worthy of a glance,” meaning a civil gaze. Both hate stares (already discussed) and ways of treating others as if they were not there can be non-person treatments. Here, Goffman is exceptionally vacuous. The groups of people he mentions—children, servants, Negros and mental patients—categorically belong to varying social situations where “non-person treatments” rely on different interactional practices and vary in their consequences.^11^ More importantly, most of the “non-person treatments” take place in focused interactions. Some practices, such as avoiding gazing beggars (Lankenau, 1999), are done to maintain anonymous order by resisting attempts to breach it. Our interest only includes practices that take place in unfocused public space. These may involve “hate stares,” but it is not yet clear if they encompass practices of “not looking.” Goffman does discuss the right, or entitlement, to civil inattention, and suggests that uncivil behavior, such as staring at others, may weaken the expectation of civil treatment. Here also, Goffman does not go all the way; he suggests that there are a set of systematic practices to deprive personhood in unfocused interactions, apart from hate stares. Displays of not seeing could work that way, and would be based on the observability of gazing (Kidwell and Reynolds, 2022). In our material, there is one case (out of about 2,000 passings) in which a person builds a dramaturgic performance of not gazing the passerby. This performance would be of interest as such, but it is not included in the set of analyzed cases of this article. All in all, not gazing as such does not seem to constitute “non-person treatment,” which fits well with the nature of civil inattention and auspicious public order being based on peripheral awareness.

### Breaches of civil inattention

So far, we have dealt with unobtrusive gaze behaviors in public space. There are also types of gaze behavior that go beyond that, breaches of civil inattention. In some instances, the passerby invests some additional attention in the object of the gaze during the passing. These cases vary in the intensity of involvement and can be discussed as separate gaze types. We will present three types of gazes in a hierarchical order, starting from “smallish” extensions and moving toward more overt breaches of civility. Throughout the section, we will try to show how the norms of civil public behavior are broken.

### Intensified gazes

The boundary between civil and uncivil attention is not clear-cut. Goffman notes that the closer the participants of gazing are, the more the intensity of gaze grows. In cases when the gaze continues just a bit longer, it becomes noticeable, even if did not establish a proper stare. Intensified gazes form a border zone between civil inattention and marked gaze contacts. The intensity of gaze is largely brought through proximity to the gaze target. The gaze may be maintained just a fraction of a second longer, so that it still prevails at a close distance; the head may also be rotated noticeably just before passing. The gaze itself may remain relatively brief. What makes intensified gazes distinct from civil inattention is the unavoidable involvement: the receiver will always be able to detect the gaze, and the illusion of privacy will be shattered as a result. In (4), the passerby gazes briefly from a further distance but then gazes again right before passing. This latter gaze gets the emphasis of the rotating head, which turns toward the experimenter, allowing direct and undisguised observation at a close distance.


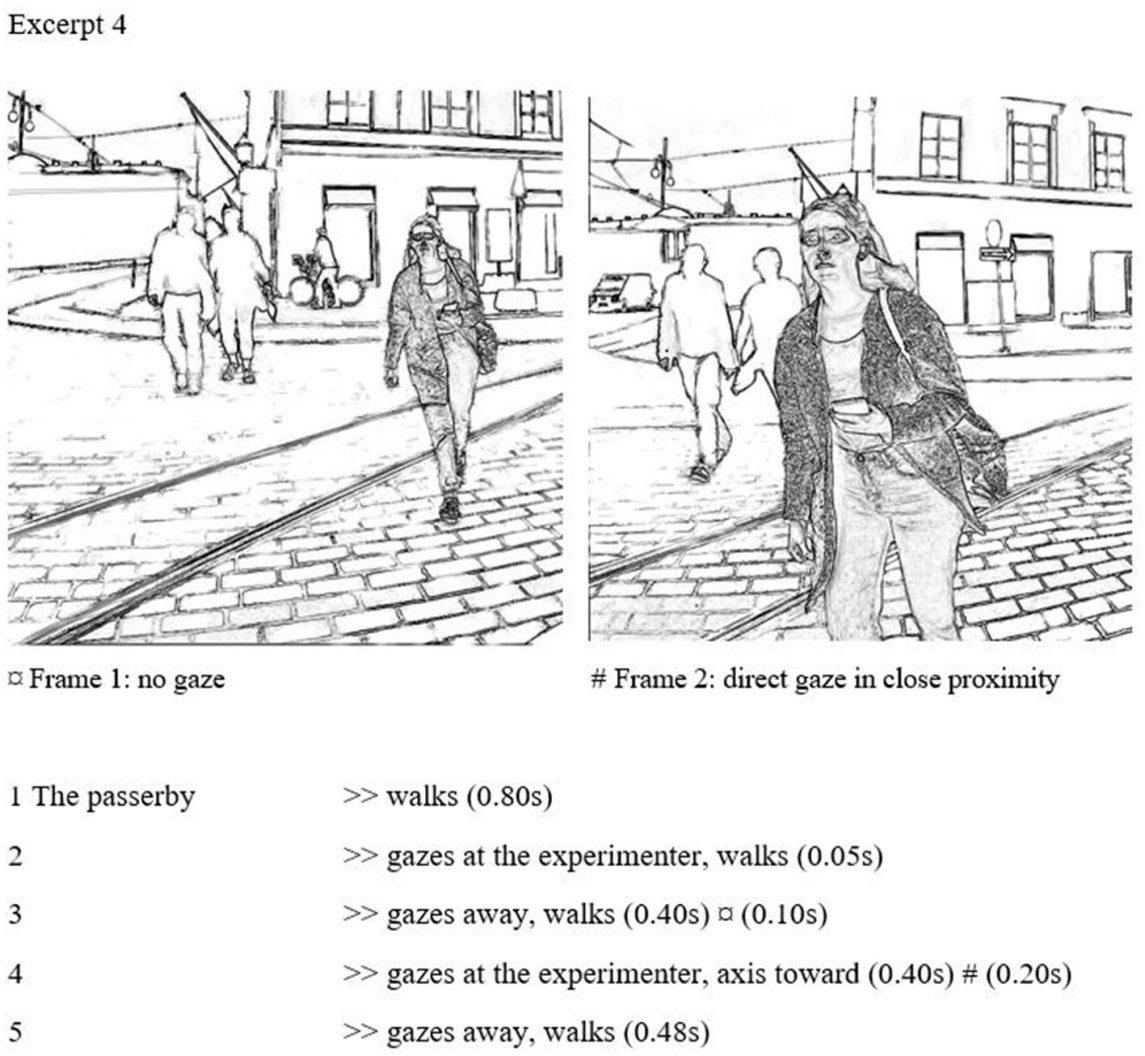


On line 2, the subject casts a micro gaze from a further distance, and then the gaze is quickly dropped (frame 1). This very brief gazing does not invite gaze contact. In contrast, the gaze right before the passing (line 4, frame 2) is direct and noticeable, although relatively brief (0.6 s). Intensity is stressed also by the proximity: the gaze is from a closer distance than the gazes in civil inattention examples. The subject is positioned right in front of the experimenter, about to pass her on the left. During the gaze, the subject's head turns slightly toward the experimenter, which adds more emphasis to the gaze. As a result, the gaze seems very direct and open. Although the gaze is not very long, the proximity and the head movement make it intense, differentiating it from civil inattention. This type of gaze is always observable by the receiver.

### Extended side gazes

Extended gazes are a type of more prominent breaches of civil inattention. In these cases, the passerby does not retract the gaze after a brief scanning but casts a longish look on a person. An extended gaze signals increased interest toward the target individual; as discussed above, extended maintaining of visual contact does something other than granting personal space. Operationalizing Goffman, we suggest that gazes closer to a second are long enough that the onlooker appears to be engaged in a focused gaze that may continue despite being (potentially) noticed.^12^ Thus, we suggest that about a second is a justifiable lower limit for an “overlong” look, something that could be called a stare that clearly breaches civility. As a further qualification, the gaze length is only an aspect that impacts its intensity and noticeability. Goffman (1963a) notes that if you are far enough away, you may “safely” look longer than civility allows at a closer proximity. The head pose further impacts how observable the gazes are; the subject may gaze either indirectly (i.e., performing a sidelong gaze from the corner of their eye) or gaze overtly with a rotation of the head toward the target. For these reasons, extended gazes are variable also in terms of their proximity and openness; hence, we cannot establish context-free absolute, precise limits for them.

Excerpt 5 is an example of an extended side gaze. The passerby approaches the experimenter on an empty street with no visual obstacles, gazes from a further distance and keeps gazing all the way until passing the experimenter completely. However, although this gaze is very long in duration, the subject maintains some level of discretion by gazing sidelong.


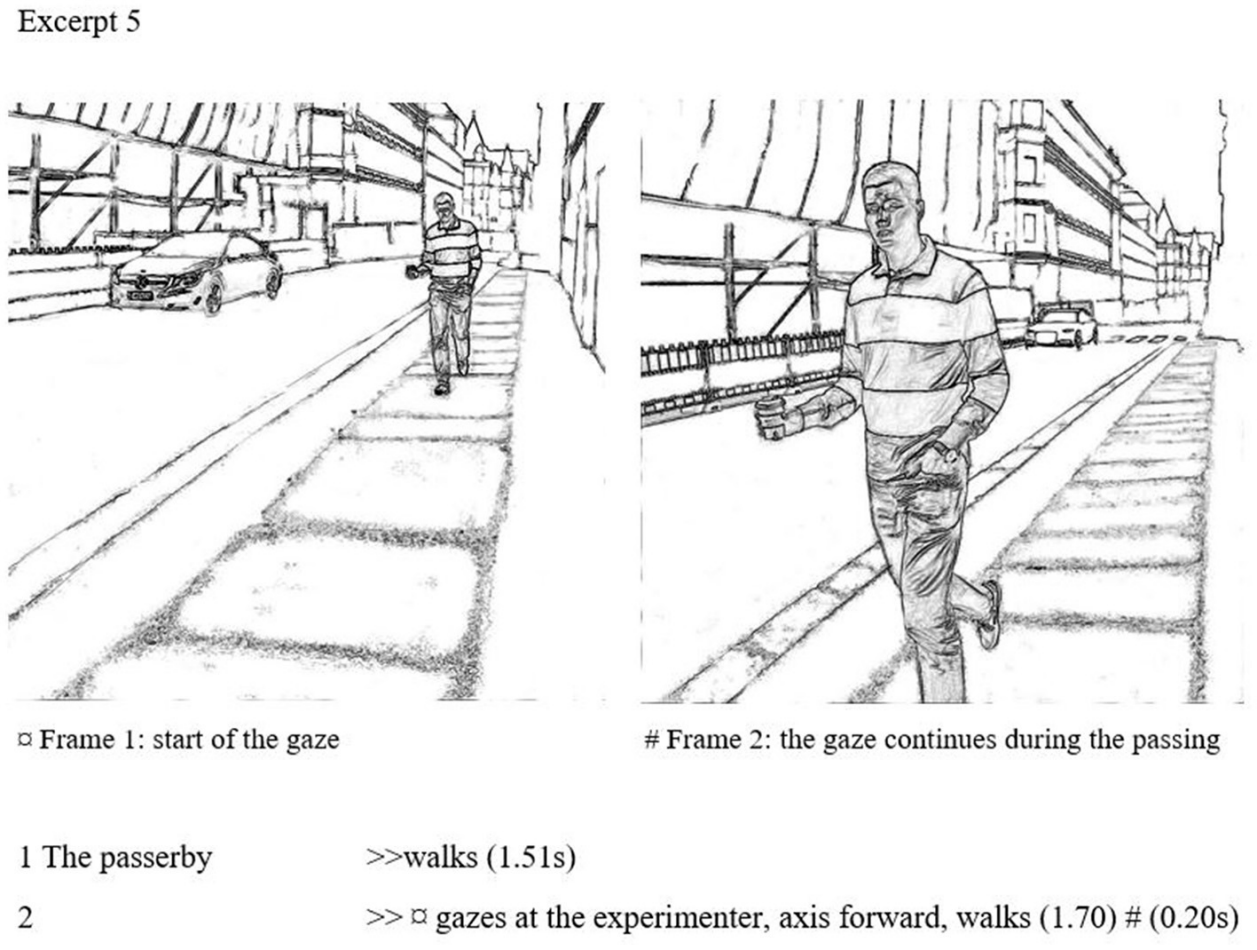


Here a gaze duration is 1.90 s, during a 3.41-s passing. Frames 1 and 2 present this long gaze in a comprehensive way. The beginning of the gaze is shown in frame 1, in which the passerby is about 5 m away from the experimenter and continues until the subject exits the screen. The mere duration of the gaze clearly oversteps the limits of civil inattention: maintaining a gaze without withdrawal for almost 2 s is rare and marked in a passing between two unacquainted passersby.

However, the gaze is set sideways, without rotating the head, which makes it partly disguised. Though discreet, the gaze is noticeable by the receiver, and far too long to be considered as civil between unacquainted persons in a public place. Moreover, it is further away from civil inattention than the intensified gaze in Example 4: both gazes continue in proximity, but the extended duration also significantly increases the intensity of involvement. This gaze is almost three times longer than the previous intensified gaze.

### Extended direct gazes

The most prominent gazes are both long in duration and emphasized further by a rotation of the head toward the experimenter. These gazes stretch far from the average gaze behavior and are clearly marked. In Excerpt 6, we present one case of an extended direct gaze. Again, the passing takes place on a relatively empty street, where the subject has clear visual access to the experimenter. While approaching the experimenter, the subject rotates the head toward her, prolonging the already prominent gaze contact.


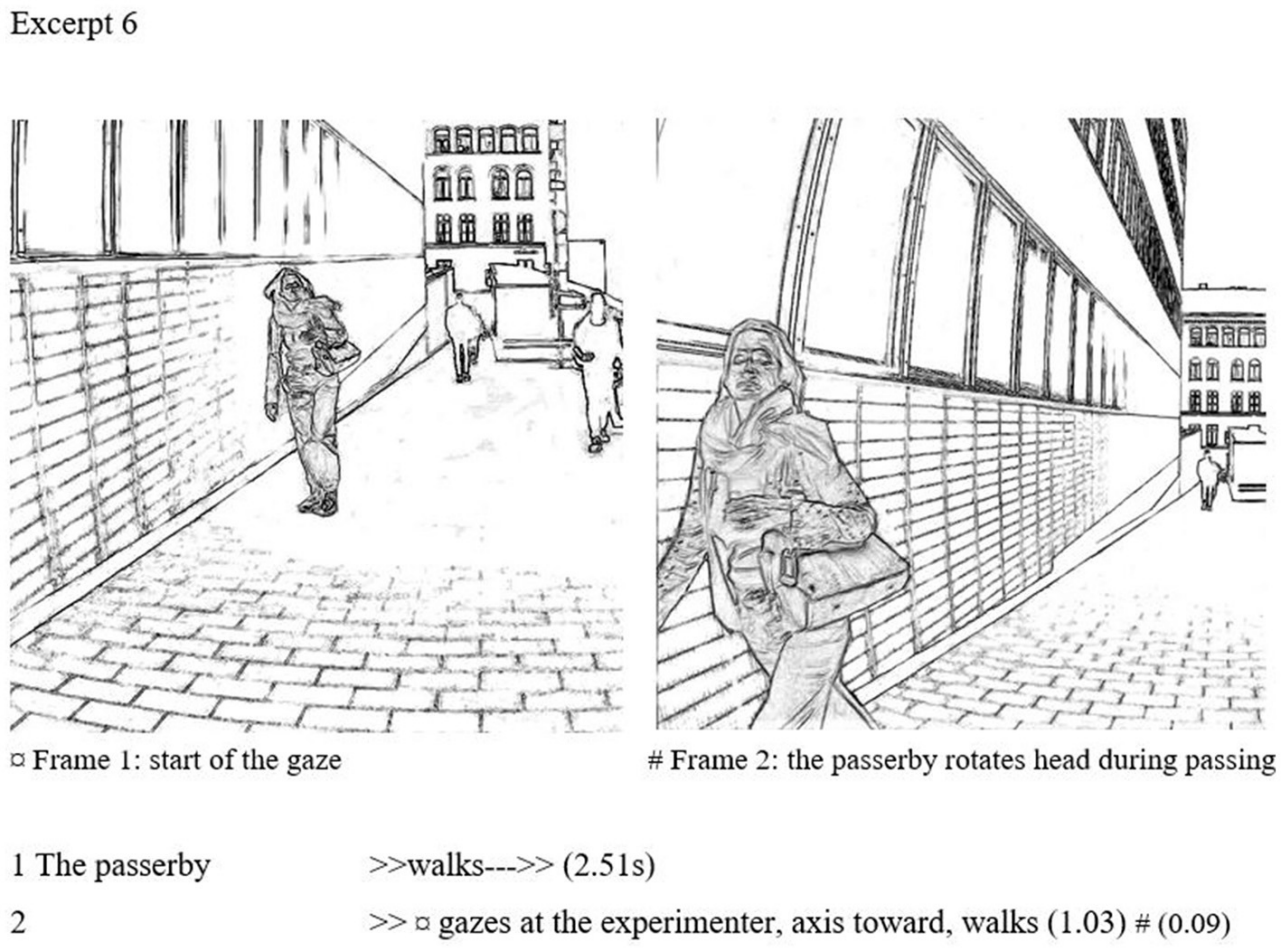


In frame 1, the gaze starts. The passerby is still relatively far away from the experimenter. In frame 2, the subject is about to pass the experimenter. The subject is turning the head quite clearly, and the position stands out even more clearly during the passing. This gaze is direct and overt, lasting for ~3 m or 1.12 s. The head rotation toward the experimenter takes place during the last 0.62 s of the gaze and remains turned during the passing. The turned head makes the gaze striking, giving the receiver a sensation of being stared at. The passerby does not disguise the gaze but stares openly.

In our categorization, an extended direct gaze is the clearest breach of civil inattention. While collecting data, the experimenter mentioned that occasionally she felt uncomfortable, and that she was able to tell that some passersby were staring at her. This type of gazing corroborates (Goffman, 1971) observations of corrective gaze behavior (i.e., sanctioning appearances that stand out from the crowd). The receiver is left wondering what provoked the stare, and what has made her accountable, which may be felt stigmatizing (Goffman, 1963b; Lamont, 2018). On the other hand, an extended gaze does not reveal the valence anchored to other features, such as facial expression and gestures. As in (6), if the passerby remains po-faced and does not engage in expressive behavior, it remains opaque whether an extended gaze demonstrates positive attention or admiration, a negative attitude or even hatred. This expressive neutrality can still separate such cases from overt uncivility, or “hate stares.”

### Summary of qualitative findings

Our data supports the claim that it is not uncommon for passersby to gaze at the recipient briefly, withdrawing the gaze before passing. The detailed video analysis allowed us to also refine the phenomenon of civil inattention. First, there appears to exist a great variation in the ways that civil inattention is performed: the duration of the gaze varies from a micro glance (0.02–0.1 s) to potentially noticeable glances (0.5 s). Also, passings without any gazing seem to be very common in an urban cityscape. Given that civil inattention is based on peripheral attention, non-gazing does not challenge it. Moreover, passersby may gaze at the recipient twice; as the second gaze is unnecessary for the navigation of the upcoming passing, it is a sign of additional interest. There are also breaches of civil inattention in the material. Gazes that are maintained just a fraction of a second longer make them intensified and noticeable, as the gaze still prevails at a closer proximity; rotation of the head may add further intensity. When a gaze lasts up to a second, it becomes clearly noticeable. Also, long stares are variable. They can be done from the corner of the eye or emphasized by gazing overtly and then rotating the head during passing, making the gazing even more noticeable. Such breaches of civil inattention break the auspicious anonymous public order.

### Causal conditions for gazing

In this section, we will present our quantitative analysis of gaze types. Through quantification we aim at building a scale for the intensity of gazes, which allows us to provide a quantitative account of gaze behavior. We then compare the gaze behavior toward recipients belonging to different social categories, operationalized with their outfits: regular Western attire, the sun hat, and the niqab. The outfits are then a causal condition for the related gaze behavior. Methodologically, we move from ethnomethodological video analysis to statistical analysis.

### Gaze scale

Our sample consists of 300 passings, 100 for each outfit and 50 for each data collection session. We will first open the quantification process before discussing the distributions of the typified gaze behavior. Initially, each individual gaze (or lack of gaze) underwent detailed scrutiny in multiple data sessions,^13^ during which we organized and reorganized the cases into separate categories. In some sense, we were trying to find a way to account the gazes for intersubjectively available actions, which would also allow their representation as distinct action types (Sacks, 1989). The procedure also ensured an inter-rater reliability of our analysis. Initially, we labeled the cases simply with the number 1, 2, or 3. Number 1 stood for “no gaze,” number 2 for “civil inattention,” and number 3 for any “breach of civil inattention.” This quickly proved insufficient. First, the cases with more than one gaze led to a new category. We then noticed inconsistency within this category, which was consequently split into two: (1) two brief gazes within the limits of civil inattention, and (2) two gazes, at least one of which included a breach of civil inattention. Later, the latter was dissolved and merged into the other “breach” categories. We noticed a pattern that there was a brief, less intense gaze from a distance and subsequently a new gaze at closer proximity. The second gaze could be either a brief, civil one or an extended, intense one. Interestingly, we found no pattern in which a lengthy gaze from a distance would be followed by a brief glance at proximity. Thus, it became possible to classify the gazes according to the type of the second gaze. As a result, we were able to propose a hierarchical scale of gazes, according to their intensity of engagement. The scale is presented in Table 1. As discussed in the qualitative analysis section above, it extends from the least to the most intense involvement: no gaze (1), civil inattention (2.1), civil inattention with two brief gazes (2.2), intensified gaze (3.1), extended side gaze (3.2), and extended direct gaze (3.3).

**Table 1:** Gaze scale.

**1**.	**No gaze**	**↓ The intensity of involvement increases**
**2.1**	**Civil inattention**	
**2.2**	**Civil inattention, two brief gazes**	
**3.1**	**Intensified gaze: short extension in length or at close range**	
**3.2**	**Extended side gaze**	
**3.3**	**Extended direct gaze**	
		**▾**


During the video analysis, the maximum duration of civil inattention (2.1) was operationalized to two steps. Counting the steps was proven to be a decent way to measure the duration of the gazes; it provides a relatively objective measurement, as the duration of steps does not vary too much between individuals. Category (2.2) for two short gazes is a subcategory of civil inattention, and therefore the criterion is similar. The intensified gaze (3.1) extends to the closer proximity of a recipient but lasts <3 steps. The intensified gaze category was initially formed as a “leftover” between civil and uncivil gazes, and it might still benefit from qualitative elaboration. The extended gaze (categories 3.2 and 3.3) is consequently three steps or more in duration. Extended gazes, moreover, are split into two separate categories based on the directness of the gaze: if the passerby turns their head to prolong the gaze, the gaze becomes visibly more noticeable and marked, compared to indirect gazing. Apart from proximity, axis (directness of facing) is a central aspect of human proxemic behavior (Watson, 1970; Conty et al., 2012). As noticed by the assistant during the experiment, some of the most direct gazes continued after the passing of our experimenter (though not captured by the video). This type of an overt—and, in a way, challenging—gaze expresses the strongest engagement. In comparison, a side gaze is less direct and does not invite involvement as clearly, even if the duration remains the same, and it does not project an extension after the pass.

Our next step consisted of refining the gaze-type categories by counting their duration. Through this we aimed at verifying the upper and lower length limits for the gaze types to prepare the data for statistical analysis. We used the Adobe Premier Pro program for all 300 cases to measure the duration of gazes with 0.01-s accuracy. The program allows examination of clips as series of frames, thereby creating a timestamp for each frame. With the maximum frame-per-second settings and an effective zooming tool, it is possible to define the exact starting and end points of gazes and gestures. Then the whole data was coded into SPSS, which allowed checking of the whole material. After a few corrections, we defined duration-based upper and lower limits for each gaze type.

Table 2 presents the numerical values for the length of gaze types. The civil inattention category (2.1) includes gazes that vary between 0.02 and 0.56 s in duration. Although all these gazes are brief, it is obvious that there is a significant difference between a 0.02-s gaze and a 0.56-s gaze. A more detailed exploration of the variation of civil inattention remains for further research. At the other end of the scale, extended gazes vary from >1.0 to 3.40 s. One second is a neat lower limit; besides being a nice, even number, the cases are clearly gathered at over 1 s or below. For the extended gaze categories, no upper limit was needed. The longest gaze is 3.40 s, which is already very prominent and stands out from the standard gaze behavior. The intensified gaze category was created by a combination of length, proximity, and head pose; therefore, its length includes variation. Moreover, in both the two brief gazes and intensified gaze categories, there are cases with more than one gaze, and the table presents the combined duration of these multiple gazes. These cases have been analyzed individually to make sure that each gaze in these categories is within the previously defined duration limits, even if the combined duration exceeds them.

**Table 2:** Duration of gaze in each category.

		**Scale (s)**	**Median (s)**	**Std. deviation**	***N***
1.	No gaze	-	-	-	95
2.1	Civil inattention	0.02–0.56	0.06	0.187	93
2.2	Two short gazes	0.07–1.05	0.56	0.340	22
3.1	Intensified gaze	0.39–1.52	0.71	0.325	25
3.2	Extended side gaze	1.01–2.96	1.92	0.519	30
3.3	Extended direct gaze	1.02–3.40	1.94	0.647	35

Duration of gazes is combined in cases with more than one gaze.

These are the categories that we will utilize in the comparative analysis. In the next two sections, we will first account for the standard gaze behavior and then proceed to the comparisons of the gaze behavior toward different social category incumbents based on their outfits.

#### Standard gaze behavior

For the standard gaze behavior, we will use the condition “regular Western outfit” as our baseline. We assume that the experimenter who does not stand out from the crowd in this outfit would receive the average number and type of gazes from passersby, although individual variations in features such as age, gender, and height might also matter. As the experimenter remained the same during each session, this individual variation does not affect the comparative results between outfits.

As presented in Table 3, the standard gaze behavior consists of minimal involvement with passersby in the cityscape. Our analysis supports Goffman's view on the salience of civil inattention in public: it forms 46% of the cases. These gazes are typically very brief and seem to appreciate the other's privacy. As a novelty, we discovered civil inattention with two short gazes, though not commonly (in total, 2% of the passings); these are still within the limits of civil inattention but form a recognizable gaze type. However, not gazing is even more common than brief gazes (49%). As discussed, it is not a sign of rudeness or avoidance but a regular type of behavior, which may indicate the relevance of peripheral attention in a cityscape, enabling passings without any gazing. No gazing does not seem to pose any social sanctioning. Peripheral vision without a focused gaze captures enough information to socially navigate and maintain a sufficient space from passersby.

**Table 3:** Standard gaze behavior (%).

**No gaze**	**49**
Civil inattention	46
Two short gazes	2
Intensified gaze	2
Extended gaze	1
Total	100

While most of the passings do not breach civil inattention, some do. Intensified gaze and extended gaze are types of gaze behavior that demonstrate an investment of additional attention. In our data, three cases out of a 100 exceeded civil inattention in some way, and only one included an extended gaze, in which the passerby gazed at the experimenter for longer than a second. Intensified gazes appear slightly more common, but still rare. It can be concluded that breaches of civil inattention take place during everyday interactions between passersby, but sparsely. An emphasized gaze may signal interest, an attempted approach, or a condemnation, but the intention of a gaze without any clear facial expression may remain undecipherable.

#### Variation of gazes according to the recipient's social appearance

Our comparative study was based on gathering similar sets of data with all three outfit conditions: unnoticeable, regular clothes; regular clothes with a remarkable sun hat and sunglasses, and a niqab with an abaya. This study design enabled comparisons of gaze behavior depending on the social appearance of the gaze receiver. In the experiment, the datasets of different conditions were collected at the same time of day, at the same location, and with the same experimenter within a 2-week period. The weather conditions were relatively standardized by not filming on rainy days. The only difference between three datasets is the visual appearance of the experimenter. Thus, the data allows a study of correlations between the visual appearance and the received gaze behavior. All in all, we chose a sample of 100 cases of each condition, making altogether 300 cases. The distribution of gazes toward social category incumbents in different conditions is shown in Table 4.

**Table 4:** Distribution of gazes according to social appearance.

	**(%)**			
	**Regular**	**Sun hat**	**Niqab**	**Total**
1. No gaze	49	28	18	31.7
2.1. Civil inattention	46	18	26	30.0
2.2. Two short gazes	2	9	8	6.3
3.1. Intensified gaze	2	14	15	10.3
3.2. Extended side gaze	0	17	13	10.0
3.3. Extended direct gaze	1	14	20	11.7
Total	100	100	100	100.0
				*N* = 300

In Table 4, the column “regular” consists of the data collected with the experimenter's own casual clothing. As presented in the previous section, practically half of the passersby did not gaze at her at all. Only 3% of the gazes broke civil inattention; all other followed the civil inattention protocol. This supports the hypothesis that minimal involvement is the standard during passings between two unacquainted passersby, either in the form of civil inattention or non-gazing. The distribution of gazing, however, differs significantly in two other conditions: when the experimenter was wearing a sun hat or a niqab with an abaya.

In the sun hat condition, most of the passersby (55%) either did not gaze at the experimenter at all or maintained civil inattention: 28% of the passings took place without any gazing and 27% involved civil inattention, including cases with two short glances. Nevertheless, the sun hat drew considerably more attention than the regular Western outfit. In total, the gazing is both more numerous and more prominent: 14% of the cases include an intensified gaze, 17% an extended side gaze, and 14% an extended direct gaze. The extended side gaze (i.e., gazing indirectly for more than 1 s) is especially prominent with the sun hat condition. It might indicate that the hat arouses curiosity, but the passersby partly disguise it by avoiding overt gazing. The relatively large amount of two short gazes (9%) supports this interpretation: this gaze type is still within the limits of civil inattention, but the passersby tend to gaze again after the first brief gaze. The sun hat may act as a novel stimulus, encouraging the passerby to pay more attention than they normally would.

The experimenter dressed in the niqab also clearly attracted more gazes than the regularly dressed gaze recipient. Only 18% of passersby gave no gaze at all. The proportion of civil inattention, however, remains significant and is slightly higher than with the sun hat condition (34%, including two short glances). Wearing a niqab, the experimenter received gazes that can be considered, on average, more open: while the amount of intensified gazing remains roughly the same (15%), the most remarkable feature in this sample is the large amount of extended direct gazes (20%). This is a high number for such an overt type of gazing: practically every fifth passerby gazed at the experimenter in a noticeable way.

We may conclude that both the niqab and the sun hat condition differ clearly from the regular Western outfit condition. The difference between the niqab and the sun hat condition is finer and does not appear to be statistically significant in our data. Since both gaze and the appearance conditions are categorical variables, we tested the significance of differences with chi-square. Both the sun hat and the niqab condition received significantly different distributions of gazes (*p* < 0.001) compared to the regular Western outfit. This verifies the hypothesis that the appearance of the experimenter did affect the gaze behavior of the passersby. However, the difference between the sun hat and the niqab is not statistically significant. Instead, it appears that any noticeable deviance from a standard appearance may draw additional attention in the form of gazing that extends beyond civil inattention, but the gazing in our sample does not appear to be motivated by ethnic or religious grounds but as recognition of a deviance from normal appearance.

## Discussion

Video analysis confirms that in public places, gaze behavior between unacquainted passersby commonly consists of brief gazing well before passing. Empirically the study supports Goffman's concept of civil inattention as the prevailing urban ritual. The extreme brevity of most civil gazes suggests that they are not open for focused mutual gaze contact, as it might not be possible within this time scale (Thorpe et al., 1996; Willis and Todorov, 2006; Conty et al., 2012). This means that civil inattention is not a reciprocal practice, as it is sometimes carelessly described (Giddens, 1990, p. 81). Instead, the rarity of focused mutual gaze contacts within civil inattention stresses the role of peripheral awareness for mobile social navigation in urban surroundings (Heath and Luff, 1992; vom Lehn et al., 2001). Peripheral awareness enables individuals to pass each other with minimal gazing or no gazing at all. Consequently, passings without gazing are not a curiosity, and they do not challenge the auspicious public order, where others do not pose an immediate threat.

As farsighted as Goffman's writings are, detailed video analysis allows us to move beyond the limitations of ethnography. First, civil inattention may be more multifaceted than suggested. Gazes that are short enough to be considered civil include a huge variation, from micro glances of 20 ms to potentially noticeable 500-ms gazes. Also, multiple gazes by passersby are common. Together these findings suggest that there may be differences in the investment of attention in the recipient already within the bounds of civil inattention. Further, moving out of a civil gaze may also be more variable than proposed. All this suggests that there may be layers of monitoring involved in the maintenance of civil public behavior that have not hitherto been sufficiently dealt with. Civil inattention may be just a gloss for a complex architecture through which agents maintain the distinction between engagements and anonymous public order.

Comparative analysis of gaze behavior toward different social category incumbents shows that there is a clear variation of gaze behavior according to the gaze recipient's appearance. The auspicious civil public order appears to be, at least to some extent, exclusive; passersby who deviate from normal appearances may not be granted the same level of civility in public areas as those who conform to the local cultural norms. The difference of gaze distribution toward “normal appearance” and “marked appearances” is statistically significant. In fact, the amount of uncivil extended gazes toward atypical appearances is 10-fold compared to normal appearances. This is a striking increase of interest. Saliently, there was no statistically significant difference between sunhat (culturally unmarked) and niqab (culturally marked) conditions. Our study shows that gaze behavior is somewhat equally attuned toward all normatively deviant atypical appearances but is not intrinsically culturally prejudiced. The lack of facial visibility may be a common element, as a cause of discomfort and additional attention, as Moors (2009) and Tarlo (2010) have proposed. We did organize an additional experiment where the comparison was made between the experimenter with or without gaze-hiding sunglasses, and the result was that the sunglasses did not affect the recipients' gaze behavior. The visibility of gaze as such does not appear to be critical for recipients; therefore, cultural factors may in any case be more salient than behavioral features of face visibility. Indeed, the niqab condition attracted the greatest amount of extended direct gazes that can be considered undisguised. These kinds of gazes may impose a threat of privacy and personal space in public, and they may be felt to be stigmatizing. Further, publicly announced negative attitudes toward minorities may open them to unhindered stance displays and result a recognition gap for them (Lamont, 2018). This said, our data did not involve any clear facial expressions of strong negative emotions (e.g., hate), and in that respect all interactions included at least an aspect of civility. We would need a more detailed analysis of intrusive gazes to unpack their socio-semiotic mechanisms to identify “hate stares” (Timmermans and Tavory, 2020). The semiotic properties of extended gazes could be explored in terms of whether the gazer's emotional state can be recognized with any intersubjective reliability, and whether the type of gazes concentrate to a certain categoric recipient, i.e., do categoric identities evoke negative emotional states, that is, hate.

The finding of the selectivity of civility in public behavior seems to have a relation to unconscious stereotypical biases. Our study seems to provide support for Implicit Association Tests (IAT), suggesting that gaze behavior is affected by unconscious biases (Banaji and Greenwald, 2013). However, it has been noted that although people almost automatically respond to stereotypes, the valence of their response may vary according to their group-based values (Arminen and Heino, 2022). It is apparent that civil inattention as a boundary mechanism that keeps engaged interactions between ratified participants and public order apart is far richer and more complex than initially perceived. It may well be that there are layers of monitoring that sweep public space as an enabling practice, the consequence of which is “civil inattention.” This opens civil inattention itself to a reverse engineering. Rather than assuming its existence, a finer granularity of analysis could reduce it to its elements. The monitoring of social space with a sweep of ultra-brief gazes may be the elementary layer of public order, invoking a variety of possible courses of action, including second gazes. The reciprocities of gaze behavior during interaction are one aspect influencing the outcome of the next actions. Whether peripheral awareness is preceding all this adds still another layer. Multilayered monitoring of social space makes the selective nature of civil inattention more understandable. This preconscious monitoring may be the selection mechanism through which the amount of invested attention is chosen. If that were the case, then there appears to exist a mechanism below conscious decisions that influences the amount of attention directed toward recipients, thereby initiating categorization of recipients (c.f. Cerulo, 2018). In that way, the social world appears to be structured at the outset rather than being a level playing field (Fiel, 2021). This formative mechanism that amounts to relational segregation may enforce anti-civil forces into the social system by imposing a set of asymmetric relations between social categories. In that way a source of anti-civil forces do not come from the outside, from the non-civil institutions, but through the boundary mechanisms that are the structuring precondition of social interaction (c.f. Alexander, 2006).

Civil inattention is a paradoxical ritual action that precludes the establishment of an engagement. It does not invite a response; only extensions of attention would invite a response, either a counter or a withdrawal (more typically). Hence, civil inattention precedes structured engaged interactions as a taken-for-granted structuring precondition of an interaction order (Brekhus, 1998). As a miniscule ritual grounding of interaction order, civil inattention forms a repetitious, mass-scale structure, which works as a boundary mechanism between focused and unfocused interactions. Paraphrasing Collins (1981), civil inattention initiates the microscopic sources for streams forming via recurring typifications relational categories providing basis for coalitions. But as civil inattention is largely based on peripheral attention, it precedes the level of events normally attended in micro sociology, a kind of neurological foundations of sociology (Cerulo, 2010). The gaze behavior in public may have an initial imprint on relational segregation, the structural effects are the aggregate outcome of gaze behavior. The granular analysis of gazing behavior may allow us to discover how the valencies of relationship are formed in face-to-face. The exploration of aggregated distribution of categorically distinct recipients may allow us to distill how categorical differences in practice build boundaries on those differences that may form relational segregation bound to lead to segmented networks as a societal effect (Fiel, 2021, p. 157). This may also be a mundane source for the persistence of social inequalities (O'Connor, 2019). Notably, gender, age-grade and race are visually easily perceptible statuses, which makes them omnirelevant categoric identifications that precede engaged interactions (Goffman, 1983). As all categoric identifications are contingent, cognitive and historically varying that would make comparative analysis of gazing behavior and the ensuing public social order salient to explore potential variation in the types and degrees of relational segregation.

## Data availability statement

The datasets presented in this article are not readily available because, restricted to use of the authors only. Requests to access the datasets should be directed to ilkka.arminen@helsinki.fi.

## Ethics statement

The research practices follow the guidelines of the Finnish National Board on Research Integrity. Although consent to participate was not requested in advance, the research is justified and could not have been carried out if the participants were asked for their consent to participate in the research. Data collection did not cause damage or harm to the participants. An ethical approval statement can be given by the review board. The studies were conducted in accordance with the local legislation and institutional requirements. Written informed consent for participation was not required from the participants or the participants' legal guardians/next of kin in accordance with the national legislation and institutional requirements.

## Author contributions

All authors listed have made a substantial, direct, and intellectual contribution to the work and approved it for publication.

## Footnotes

^1^Together the abaya and niqab comprise what is commonly known as the burkha.

^2^We apologize for the inappropriate language. Notably, J. H. Griffin was a citizen rights activist and critical toward all racial discrimination.

^3^The sun hat was from a global online store, and it did not represent any known ethnic or religious group, either for us or in its marketing.

^4^The total population in Finland is above 5.5 million. There is no official number of Muslims, because the majority of Finnish Muslims do not belong to a registered religious community.

^5^Konttori's observation of the rarity of niqabs is certainly true in the city center of [city]. In certain districts, Muslim population percentages are relatively high, and traditional Muslim attires are not uncommon there.

^6^The transcript begins from the precise moment when the passerby becomes visible in the video recording and ends at their disappearance after passing. Here the total length of the episode is 3.65 s.

^7^The anonymized stills with a limited number of pixels do not really enable access to the gaze direction of subtle, brief gazes where the head is not visibly rotated. Due to the procedure where consent could not be requested in advance or where contacting more than 2,000 passersby afterwards would have not been feasible, anonymization remains a necessity. In any case, the embodied aspects of alleged gazes can be shown with anonymized stills.

^8^Our procedure does not allow us to explore reciprocity of gaze. Basically, we posit that a half-second gaze is long enough to be noticed but not yet a stare.

^9^We aim to develop the analysis further by comparing brief and longer cases of “civil inattention,” but more on that in another publication.

^10^Our data sample includes 13 passersby who were conversing. The engagement in the conversation does not as such exclude the possibility of monitoring the environment. Some parties in the conversation do gaze at the experimenter. Such cases are excluded from the data only when visibility of their eyes was hindered by others (i.e., a crowd or a group ahead of the passerby).

^11^Among others, we could differentiate the attention deficit toward the assisting staff, how “adult talk” is established by not attending children, how institutional care is organized, etc. Many of these are based on institutionalized arrangements that presuppose engagement. Expressions of hate are an exception to that.

^12^The counter argument is that the gaze became extended, as the experimenter had withheld attention from the passerby. Our limitation is that we do not have data of the experimenter's gaze movements. We just assume that the experimenter has scanned the passersby around within a second. Reciprocity of gazes would benefit from further research.

^13^Authors were regularly present at data sessions, accompanied occasionally by a prospective research student who had done a master's thesis on gaze behavior. In the sessions, we discussed how we saw the gazes in the video data. All parties in the sessions were under the confidentiality clause.
